# Epigenetic roles of PIWI proteins and piRNAs in colorectal cancer

**DOI:** 10.1186/s12935-021-02034-3

**Published:** 2021-06-30

**Authors:** Fatemeh Sadoughi, Seyyed Mehdi Mirhashemi, Zatollah Asemi

**Affiliations:** 1 Research Center for Biochemistry and Nutrition in Metabolic Diseases, Institute for Basic Sciences, Kashan University of Medical Sciences, Kashan, I.R. of Iran; 2 Metabolic Diseases Research Center, Research Institute for Prevention of Non-Communicable Diseases, Qazvin University of Medical Sciences, Qazvin, Iran

**Keywords:** PiRNA, PIWI protein, Epigenetic, Colorectal cancer

## Abstract

Small non‐coding RNAs (sncRNAs) are a subgroup of non‐coding RNAs, with less than 200 nucleotides length and no potential for coding proteins. PiRNAs, a member of sncRNAs, were first discovered more than a decade ago and have attracted researcher’s attention because of their gene regulatory function both in the nucleus and in the cytoplasm. Recent investigations have found that the abnormal expression of these sncRNAs is involved in many human diseases, including cancers. Colorectal cancer (CRC), as a common gastrointestinal malignancy, is one of the important causes of cancer‐related deaths through the entire world and appears to be a consequence of mutation in the genome and epigenetic alterations. The aim of this review is to realize whether there is a relationship between CRC and piRNAs or not.

## Introduction

Every function executed in our cells is conducted and monitored by a variety of genes which are altogether making up our genome. For years, it was believed that 98% of DNA was “junk” because of the non-coding parts but today, lines of researches have proven that these parts of DNA have some biological functions, as well. In our knowledge, more than 70% of the human genome is actively transcribed but protein-coding genes make only 1–2% of the genome, and the major group of transcripts is noncoding RNAs (ncRNAs). Non-coding genes contain introns, pseudogenes, repeat sequences, and cis/trans-regulatory elements and they can operate as RNAs without any translation [1, 2]. Recent investigations on RNAs have suggested that non-coding RNAs (ncRNAs) make 99% of the total RNA content in every cell [3]. Additionally, some researches showed that a part of ncRNAs are functional [4]. NcRNAs are categorized by their function into three groups: housekeeping ncRNAs (like ribosomal RNAs (rRNAs), transfer RNAs (tRNAs), and regulatory ncRNAs. Also, regulatory ncRNAs can be divided into two subtypes: small non-coding RNAs with less than 200 nucleotides and long non-coding RNAs with more than 200 nucleotides. MicroRNAs (miRNAs), P-Element induced wimpy testis (PIWI)—interacting RNA (piRNAs), small interfering RNA (siRNAs), and small nucleolar RNA (snoRNA) are four kinds of RNAs classified as small non-coding RNAs which differ from each other in many aspects including their biogenesis pathways [5–7].

As a member of sncRNAs, piRNAs have 24 to 32 nucleotides. A trial investigating how Stellate protein-coding gene repeats are silenced in the Drosophila melanogaster male germ line, helped to discover the existence of piRNAs for the first time [8]. These RNAs were called repeat-associated small interfering RNAs (rasiRNAs) at first, but then, after revealing their interaction with PIWI proteins they were named piRNAs [9, 10]. Recent studies have shown that sncRNAs can take part in the regulation of gene expression and thereby, they can be key players in many physiological and pathological activities in the body. This feature is possible through RNA interference, RNA modification, and spliceosomal involvement and as a result, they might be involved in several diseases such as cancer [2, 6, 11]. piRNAs, as a class of small noncoding RNAs, also are participating in regulation of genes and proteins; but generally, it’s not easy to specify their exact functions; because not only there is a great variety in piRNAs’ sequences but also their activities can be different between species [12]. Other functions of these RNAs include silencing of transposon elements (TEs), epigenetic regulation, reorganization of the genome, spermatogenesis, and germ stem-cell maintenance [5].

In addition, PIWI proteins enhance the methylation of DNA and thereby, operate on the chromatin level and they can also compose some histone marks which repress the transcription of TEs [1]. PIWI proteins are a family of proteins encoded by the *Drosophila* piwi gene and are identified in gonad and germline development along with transposon silencing. Both mice and *Drosophila* are identified to express three classes of PIWI proteins while humans are able to express four types: HIWI, HILI, HIWI2, and PIWIL3 [13].

Some of these properties can provide an idea that piRNAs are associated with some cancer hallmarks containing cell proliferation, apoptosis, metastasis, and invasion [5].

Colorectal cancer (CRC) is a major cause of morbidity and mortality in both men and women around the world [14]. According to world cancer research fund, this cancer is listed as the third common cancer after lung and breast cancer with 1.8 million new cases reported in 2018 [15]. Moreover, developed countries have a higher incidence rate for CRC. Possible risk factors for this kind of cancer are overweight, extravagant alcohol drinking, having a CRC family history, inflammatory bowel disease (IBD), cigarette-smoking, and consuming processed red meat [16, 17]. In contrast, there are also some factors which can cause a reduction in CRC risk such as physical exercise, hormone therapy in postmenopausal women, and aspirin/NSAID use, as well as, fruit and vegetable consumption [18]. In addition, some studies declared a greater CRC risk for men than women [19, 20].

This review is an attempt for providing more effective diagnostic and/or prognostic and therapeutic approaches for decreasing the global burden of colorectal cancer and preventing more patients from suffering.

## PiRNAs biogenesis

According to investigations in this field, piRNAs are classified into three subclasses based on the variety of origins they are derived from. Transposon-derived, mRNA-derived, and lncRNA-derived are these subclasses. The first type of these RNAs is composed of the sense piRNAs along with the antisense ones. The second type is processed and originate from the 3’ untranslated regions (UTRs) while the third type is originated from the whole transcript [21]. The initiation of piRNA biogenesis is conducted by Pol II which transcribes these RNAs from their clusters mostly observed in pericentromeric and telomeric heterochromatin regions [22]. In mammalian, there are two class of single-strand clusters known for piRNAs based on the direction of their transcription: unidirectional and bidirectional [22, 23]. After transcription, piRNA precursors ought to be transferred into the cytoplasm in order to go through some alterations. This transportation is confirmed to be possible by Nxf1 and Nxt1 in *Drosophila* [24].

**Fig. 1:**
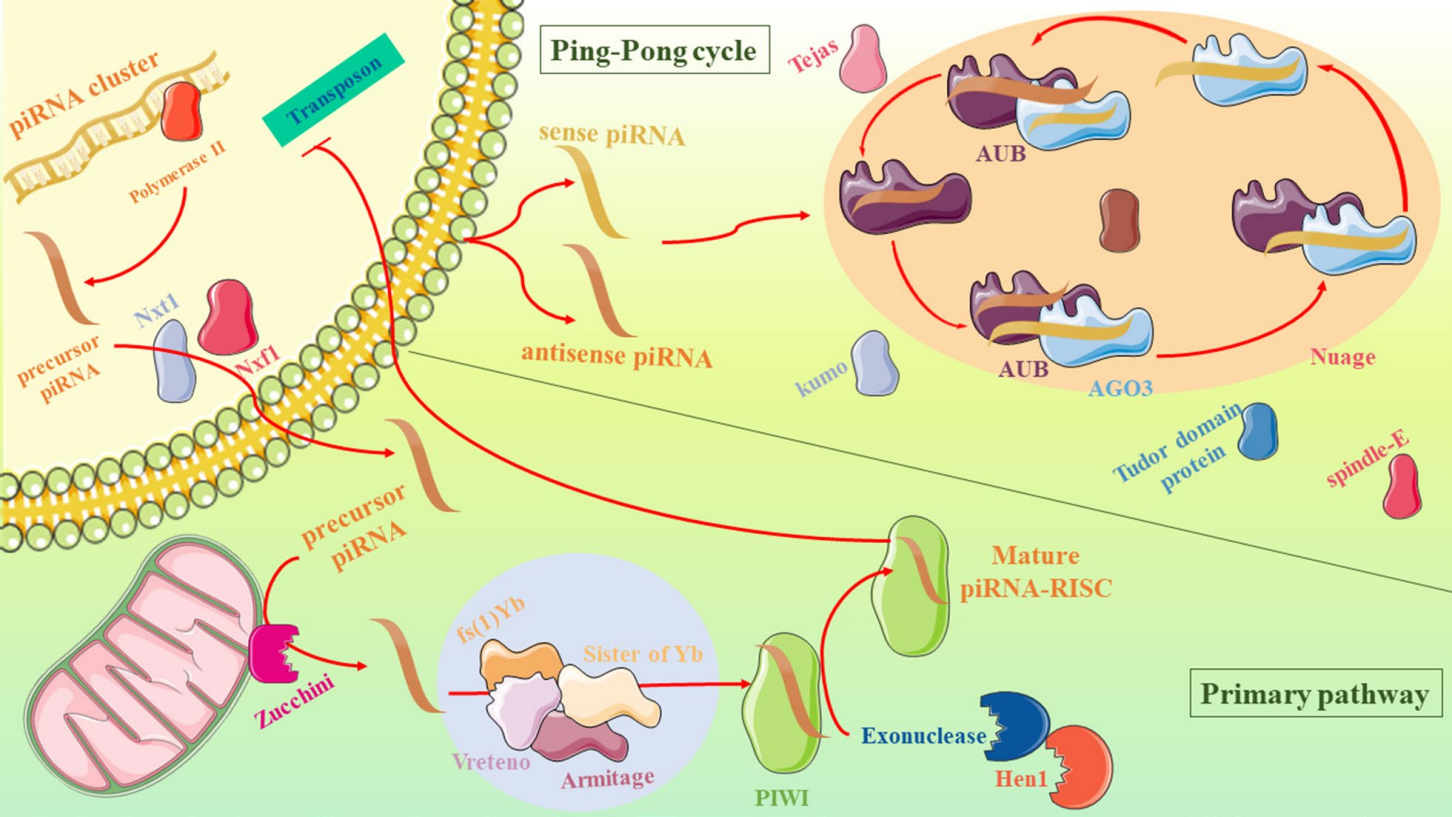
piRNA biogenesis. After piRNA transcription, their precursors are transferred into the cytoplasm in order to become mature through either the primary pathway or the ping-pong cycle

One of the reasons that piRNA is quite different from other types of sncRNAs is that its biogenesis is managed independent from RNase III Dicer, an enzyme involved in the biogenesis of other sncRNAs [23, 25]. In contrast to the several differences between piRNAs and other types of sncRNAs, post-transcriptional processes are common between these RNAs which are required for piRNAs to become mature. piRNA maturation is possible through two different mechanisms after transcription: the primary processing pathway and the Ping-Pong amplification loop (Fig. 1) [26]. In our knowledge, both of these pathways are essential for creation a powerful defense against the transposons [8]. However, the ping-pong pathway is utilized only in germline cells when the former pathway is detected in both germline and somatic cells [13].

## The primary processing pathway

There is limited evidence regarding the details of this pathway (especially in mammalians) and there is a great need for further investigations in this field.

In Drosophila, the primary mechanism is executed by an enzyme termed ribo-endonuclease Zucchini which cleaves the initial transcript of piRNA [27]. The location of this enzyme is revealed to be on the mitochondrial surface. In the following steps of this pathway some granules named Yb bodies are participating which encompass fs(1)Yb (Yb), Armitage (Armi), Vreteno (Vret), and Sister of Yb (SoYb) [28–30]. After that, the 3’portion is merged with PIWI proteins and then, its size is reduced to a final length by a 3’ to 5’ exonuclease. The hydroxyl group of the 3’ end of this piRNA ought to be methylated as a result of a methyltransferase activity named Hen1. While all these are happening, the other end of the transcript (5’end) represents a strong bias for uridine residue [31]. At the end of this process, the piRNA with the final length binds to PIWI proteins in order to form a piRNA-PIWI complex. This complex has the responsibility of activating the silencer machinery and thereby, inhibiting the transcription of its target gene, but all this is only possible if this complex travels back to the nucleus. Through this mechanism, piRNAs are able to install transcriptionally silent heterochromatin and by means of that they can affect TEs [32].

## The Ping-Pong amplification loop

The importance of this mechanism is related to the necessity of amplifying primarily generated piRNAs located in the cytoplasm [31]. Generally, there are three subtypes of Argonaute protein family: AUB, AGO3 and PIWI [33] which AUB and AGO3 take part in the Ping-Pong mechanism in spite of the primary synthesis. After binding of antisense piRNAs to AUB, a complex is formed which is known as piRNA-AUB complex. AUB uses its slicer feature on the piRNA and provides a cleaved piRNA which would act as a substrate for AGO3. For continuing the cycle, a piRNA-AGO3 complex is formed in order to target the antisense RNAs and thereby, provide substrates for AUB. To summarize, piRNA molecules can be built in the cytoplasm from a substrate provided by another functional piRNA’s product based on an amplification mechanism [22, 34]. Interestingly, all of these events are occurring in a perinuclear structure named nuage. Furthermore, previous researches indicate a role for Zucchini and Nibbler (Nbr), a 3′-to-5′ exonuclease, in this process which are mostly responsible for the 3′ end formation of secondary piRNAs [35, 36]. In addition, there are some other ingredients which aid the production of secondary piRNAs; for instance, vasa, tejas, kumo, *spindle-E, and* Tudor domain proteins [37–39].

This pathway is beneficial for not only amplifying piRNAs but also for silencing the TEs [22].

Accumulative evidence indicates that in mice, piRNA biogenesis through spermatogenesis is not related to this mechanism. However, signs of Ping-Pong are mostly observed in zebrafish, melanogaster, and very primitive animals like sponges [40].

## Functions of PIWI proteins and their associated RNAs

A number of functions are attributed to these non-coding RNAs which are discussed briefly in this section.

### Silencing of transposons or TEs

In most eukaryotes, RNA interfering (RNAi) or RNA silencing appears to be one of the essential pathways in gene regulation. RNA silencing pathway is based on the creation of small RNAs with 20–31 nucleotides; This small RNA induces the formation of a silencing complex which is called RISC (RNA-induced silencing complex) [1]. The task of piRNAs on transposon silencing is managed by the RISK complex. This complex binds to PIWI proteins and thereby, leads them to their transposon target and that is the reason why an elevation in transposon expression can be a result of the reduced or absent gene expression of PIWI proteins [41]. In our knowledge, TEs are high potent DNA sequences known for causing harmful effects on their host [42]. TEs’ damaging impacts rely on their roles in genomic instability and diversity [43, 44], gene dysregulation, harmful mutations, and chromosome rearrangement [45]. The non-long terminal repeat (non-LTR) TEs can be divided into two groups encompassing the long-interspersed elements (LINEs) and the short-interspersed ones (SINEs). Breast, colon, and ovarian cancers as well as leukemias are some diseases in which non-LTR families like L1, SVA, and Alu are involved [46].

piRNA-RISC complex silences these mobile sequences through different mechanisms including chromatin remodeling, histone modification, and targeting the RNAs transcribed from TE loci [47–49]. One of the mechanisms is managed through histone 3 lysine 9 (H3K9me3). Methylation of H3K9me3 on chromatin is one of the reasons why heterochromatin is formed in loci of transposons [50]. Heterochromatin formation can also be the result of the binding of Piwi proteins to both piRNAs and Panoramix/Silencio (Panx). Panx is a cofactor for Piwi which makes the heterochromatinization possible through recruiting the histone modification enzymes [51]. Linker histone H1 is mediating the chromatin remodeling function of piRNAs. This histone is involved in both chromatin three-dimensional structure and transposon expression [52].

### Epigenetic activating

Explorations on the somatic cells of *Drosophila* revealed that PIWI proteins and their associated piRNAs are participating in epigenetic processes. PIWI and piRNA function together as a complex when are bind to each other. The role of this complex is sequence-recognition which calls up the effectors of epigenetic including heterochromatin protein 1a (HP1a) to a definite site of genome and by that means, accomplishes epigenetic regulation [53].

PIWI-RNA epigenetic activator role was well-understood when it was discovered that the expression of 3R-TAS requires PIWI in a dose-dependent manner. 3R-TAS is a telomere-associated sequence locating on the right arm of chromosome 3. PIWI by the means of binding to both 3R-TAS and a piRNA mapped to 3R-TAS, executes its function. Any mutation in PIWI results in losing euchromatic histone modifications, which is an important mechanism in regulating the gene expression [54]. In addition, another mechanism is how piRNAs conscript PIWI proteins and H3K9me3 to the promoter region to create large complexes which conduct transcriptional silencing. These complexes cover transcriptional start sites or TSS and cause the inhibition of RNA polymerase II to recognize the TSS which results in silencing the expression of the target gene [55].

### Gene and protein regulator

Another functionality of this non-coding RNAs is how they can act as a host gene and a regulator of protein. For instance, piR_015520 is in intron 1 of the human melatonin receptor 1A gene which its expression is dysregulated in cancerous cells of prostate. PiR_015520 has been observed to have negative regulating effects on the expression of melatonin receptor 1A gene (MTNR1A) by binding to its genomic region. In addition, overexpression of piR_015520 results in a repression of MTNR1A expression in a concentration-dependent manner [56]. This information revealed that modifications in the levels of piRNA expression could impact on the expression levels of the gene where the piRNA is settled and this can raise our understanding of piRNAs as an expression regulator.

### Other functions

There are also some other roles that can be attributed to piRNAs such as buffering against phenotypic variations and genome rearrangement. Genome rearrangement of somatic elimination is a process that occurs in the zygotic genome of the daughter cells due to the production of a new and partial somatic genome which will be a replacement for parental germ line genome. This replacement hinders the transcription of parental germ line genome in the result of macronuclei after conjugation [57]. Moreover, piRNAs also take part in spermatogenesis, germ stem cell maintenance, and etc. [5]. It is obvious that piRNAs have widespread roles in different process of our cells but still, further investigations might give us novel insights on their functions.

## The role of piRNAs and PIWI proteins in cancer

One of the differences between piRNAs and miRNAs is that piRNAs are not complementary to the mRNA of potential target genes. This shows that this kind of RNAs may have a role in epigenetic regulation instead of post-transcriptional regulation for controlling different biologic incidents such as cancer [58, 59]. Epigenetic global alterations of cancers include DNA hypomethylation, histones hypoacetylation, and gene-specific DNA hypermethylation which lead to oncogene activation and tumor suppressor silencing [60, 61]. In general, the profile of the expressed piRNAs in a particular tissue plays a role in tissue identification by providing a unique signature for that tissue. PiRNAs regulate genes epigenetically and so they determine how many genes should be active at a time in a specific cell [62, 63]. Consequently, these RNAs can also be known as cancer-specific signatures because, in cancer cells, global hypomethylation and focal hypermethylation occurs and thereby, piRNAs are expressed abnormally in cancer cells [64, 65]. Some research has shown that despite of the wide involvement of piRNAs in cancer cells, only a small amount of them is expressed in somatic cells [66]. Overall, Martinez et al. [66] revealed that piRNAs have some functions in cell proliferation, apoptosis, metastasis, and invasion. Moreover, the family of PIWI proteins has some pivotal roles in self-renewal mechanism of stem and germ cells, and silencing of the RNA and translational regulation in diverse organisms [67–69]. This family contains four subtypes which any of them has a different role in cancer cells (PIWIL1 or HIWI, PIWIL2, PIWIL3, and PIWIL4) [70]. In the next sections, we are going to discuss the role of piRNAs as well as PIWI proteins in cancer cells.

### Cell proliferation and apoptosis

Several studies are conducted on different kinds of cancers to show that piRNAs can cause the inhibition of cell proliferation in cancer cells. Chu et al. [71] found that Over-expression of piRABC (a kind of piRNA) up-regulated the TNFSF4 protein and thereby, caused the inhibition of bladder cancer cell proliferation, colony formation, and also promotion of cell apoptosis. Yan et al. [72] investigated piRNA-823 in multiple myeloma patients (MM) and observed a reduction in regulators of cell cycle and the expression of proteins involved in apoptosis and the inhibition of tumorigenicity in vivo and in vitro. This trial also revealed that piRNA-823 can also reduce global methylation in DNA and re-expression of a methylation-silenced tumor suppressor. Furthermore, Jacobs et al. [73] investigated the role of piR-598 in glioma and found that this piRNA effects cell survival and increases the viability of glioma cells and the formation of the colony and as a result, it aggressively elevates cell proliferation. Moreover, also HIWI or PIWIL1 [74–81], PIWIL2 [82, 83], and PIWIL4 [84, 85] (members of PIWI subfamily) have been proven to play important roles in cell proliferation and apoptosis in several types of cancers.

### Cancer cell metastasis and invasion

Some researchers have focused on the roles of piRNAs in some cancers such as breast cancer [86–88], gastric cancer [89, 90], clear cell renal cell carcinoma (ccRCC) [91], and hepatocellular carcinoma [92] and their works are great evidence to prove that the piRNAs have the potential effects to block the metastasis and invasion of cancer cells. In our knowledge, blocking cancer metastasis in different types of cancers is done by diverse piRNAs but overall, we can say that all these piRNAs are doing their duty by two mechanisms: gene methylation and AKT pathway phosphorylation (which the second mechanism is only reported in hepatocellular carcinoma). In addition, some other studies have demonstrated that some members of the PIWI subfamily can influence metastasis and invasion in cancer cells by regulating MMP-2 and MMP-9, although further trials are needed for understanding the exact mechanism. A line of research on on HIWI has revealed a relationship between this protein and cancers such as epithelial ovarian cancer [93], glioma [80], and hepatocellular carcinoma [75]. Moreover, PIWIL2 [94] and PIWIL4 [84] also has impacts on metastasis (particularly PIWIL2 which has a role in colon cancer). Taking together, this evidence revealed that piRNAs and PIWI proteins have crucial roles in inhibiting another cancer hallmark: metastasis.

## PiRNA and CRC

Growing evidence revealed that colorectal cancer is a heterogeneous disorder. Mutations of the genome and epigenetic alterations [containing DNA methylation, histone modification and non‐coding RNAs (ncRNAs)] can activate oncogenes and inactivate tumor-suppressor genes and thereby, cause colorectal cancer [95–99]. Currently, some dysregulated piRNAs have been found in tumor tissues. In a general manner, gene expression, epigenetic processes, differentiation, proliferation, migration, and apoptosis of the cells, transcriptional regulation, post-transcriptional regulation, organ regeneration, and human disorders are the fields in which piRNAs are involved [100–103]. In the past few years, the functional mechanism of piRNAs in cancers has been considered and most of these studies suggested an epigenetic regulation role for these non-coding RNAs. Furthermore, a few studies also discovered that piRNAs regulate mRNA expression through binding to the 3′UTR of mRNAs. A recent study reported that piRNAs are able to bind to introns of a pre-mRNA and their binding results in the deterioration of targeted pre-mRNA through nuclear exosomes [104]. On top of that, Watanabe, et al. [105] indicated that piRNAs may suppress the expression levels of mRNAs by harboring transposon sequence in the 3’UTR or 5’UTR region. In addition, piRNAs may also function as natural antisense molecules which bind to CD regions of genes and thereby, they target these genes or target 3’UTR by acting as siRNAs [26, 105–107].

When all of these studies focused on the regulation of gene expression, Yin et al. [108] surprisingly found that piRNAs regulated the post‐translational alteration and activity of HSF1 via interaction with this protein. This study gave us a new perception about the way that piRNAs function through. In CRC, involved piRNAs are including piR‐651, piR‐54,878, piR59056, piR‐62,701, piR‐823, piR‐015,551, piR-54265, and piR-1245 which all are summarized in Table 1. Generally, STAT3 pathway is an oncogenic signaling that causes anti-apoptotic and pro-metastatic effects [109, 110]. For the first time, an investigation has revealed that piR-54265 expression and its interaction with PIWIL2 protein mediates the formation of PIWIL2/STAT3/p-SRC complex activating STAT3 by phosphorylation. This study suggested that over-expression and/or abnormal activation of STAT3 are enhanced by piR-54265 [111]. In addition, 85.7% sensitivity and 65.1% specificity of this piRNA in CRC diagnosis is suggesting that is has the potential to take the place of current diagnostic markers [112]. Mai et al. [111] also found that this piRNA can also be a new therapeutic target for CRC and it can be used to predict the response of cancer cells to chemotherapy in CRC patients. In another study, Weng et al. [113] identified 9 cancer-related genes (including ATF3, BTG1, DUSP1, FAS, NFKBIA, UPP1, SESN2, TP53INP1 and MDX1) which were functionally relevant to CRC. These genes had functions in key tumor suppressive pathways and their expression was associated with piR-1245 expression. The authors concluded that this piRNA can be considered as a prognostic biomarker in CRC.

**Table 1:** Experimental studies that investigated the role of piRNAs in colorectal cancer

PiRNA	Model	Expression	Function	References
PiR-823	In vitro	Up-regulated	Associated with the expression of HSP family	[108]
PiR-54265	In vitro	Up-regulated	Activating STAT3 signaling	[111]
PiR-651	In vitro	Up-regulated	Associated with T stage, metastasis	[91]
PiR‐54,878	In vitro	Up-regulated	Associated with recurrence-free survival	[90]
PiR-59056	In vitro	Up-regulated	Associated with recurrence-free survival	[90]
PiR-62701	In vitro	Up-regulated	Associated with recurrence-free survival	[90]
PiR-015551	In vitro	Down-regulated	Associated with LNC00964-3 expression level	[71]
PiR-1245	In vitro	Down-regulated	Associated with advanced and metastatic disease	[113]

PiR-823 is one of the most important piRNAs because of its roles in diverse types of cancer. According to the results of a research, piR‐823 elevated the amounts of HSF1 that is a common transcriptional factor for heat shock proteins (HSPs) family and by that, it enhances the expression of all of the HSP family except for HSP90 [114]. HSPs are classified into four groups including HSP27, HSP60, HSP70 and HSP90 [115]. This protein family is an important factor in several different cancers (including CRC) because of their association with an increased rate of cell proliferation, decreased apoptosis index, malignancy and poor prognosis [116–118]. Moreover, this RNA is increased expression is related to the advanced stages of CRC with 83.3% sensitivity and 89.3% specificity [119]. Similarly, increased serum levels of piR-24000 is also detected to be associated with CRC metastasis and higher stages [120].

In addition, Koduru et al. [121] did some comprehensive analysis on ncRNAs and they identified that there are some differences between the expression of these RNAs in different stages of CRC. Their result identified six piRNAs with statistically significant expression; Two piRNAs with upregulation in both tumor and metastasis groups; three up-regulated piRNA (including piR-hsa-25447, piR-hsa-23992, and piR-hsa-1043) and a down-regulated one (piR-hsa-28876) in tumor versus benign group; and in the metastasis versus benign group there were 22 up-regulated piRNA and 5 down-regulated ones. piR-5937 and piR-28876 are also two other piRNAs which are approved to be deregulated in CRC patients in comparison to healthy individuals [122].

Interestingly, a recent study shows that piRNAs are also useful for an earlier detection. piR-020619 and piR-020450 are observed to be increased even in early stages of CRC with a small sized tumor. Furthermore, these piRNAs are not detected in other cancer patients and therefore, they are specific to CRC [123].

According to the expression profiles of cancer-type-specific piRNAs we think that being a potential diagnostic biomarker for cancer might be a feature of piRNAs which have been ignored. Besides, piRNAs have some characteristics which make them a proper candidate for being a prognostic biomarker for cancer. PiRNAs are small molecules so they can’t be degraded so easily despite the long RNAs and also this feature allows them to pass through the membrane of the cell easier than other types. Therefore, piRNAs are more detectable in samples obtained from cancer patients [5].

## PIWI proteins and CRC

To date, there is a small amount of examination evaluating the expression of PIWI proteins in CRC. HIWI the first member of this family which has an important duty in regulating the renewal of stem cells has been shown to have over-expressed in some types of cancers including CRC [124]. Yang et al. [125] confirmed that there is an over-expression of HIWI in CRC leading to an enhancement in global DNA methylation and thereby, promoting the proliferation of CRC cells. In addition, Zeng et al. [126] found that HIWI can also be a biomarker for the prognosis of CRC particularly in patients at an early stage of disease or for whom without any lymph node metastasis. Moreover, a study about PIWIL2 demonstrated that PIWIL2, as well as PIWIL1, can be a biomarker for CRC because of its higher expression in this kind of cancer [127]. Another immunohistochemical trial revealed that all of the members of this family are overexpressed in CRC and they also found that PIWIL2 and PIWIL4 can be used as biomarkers; the former for early diagnosis and the latter for advanced tumors with distant metastasis [128]. In addition, a research found that the combination of sncRNAa with a nano-size polymer carrier might be a new therapeutic tool for colorectal cancer [129].

## Conclusions

**Fig. 2:**
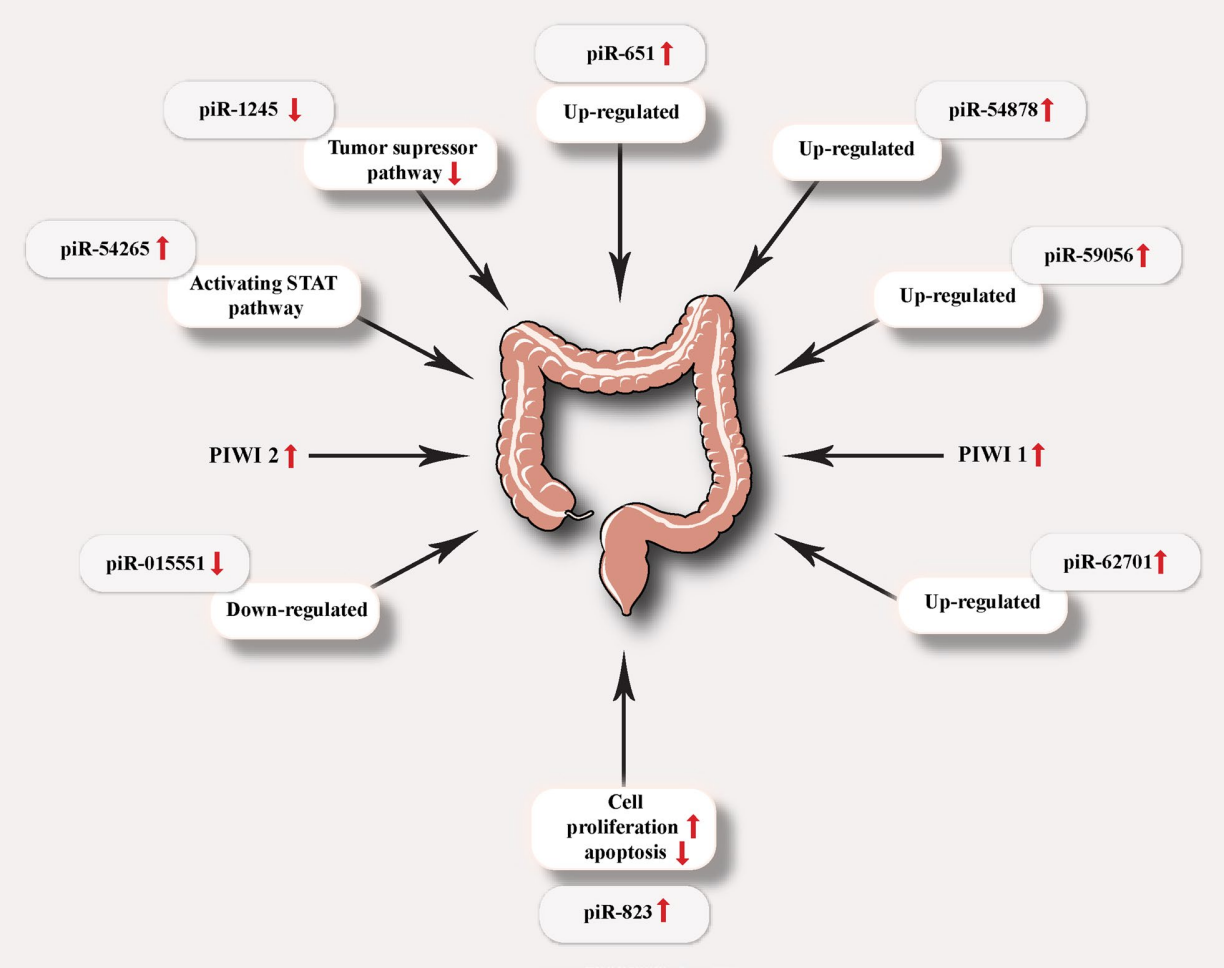
Factors related to PIWI proteins and piRNAs in colorectal cancer

PiRNAs are a group of small-noncoding RNAs which function by forming complexes with PIWI protein family. They provide a system that protects the genome against the expression of transposon elements. After revealing their role in inducing apoptosis and preventing the cell proliferation, this thought was formed that they may also be involved in cancer. Till now, several researches are done due to find a therapeutic or diagnostic approach relying on the amounts of piRNAs in different types of cancer. Colorectal cancer, considering its importance because of the high number of reported cases annually, is one of this cancer on which a few investigations has been conducted. Accumulative evidence expresses that PIWI proteins and their related RNAs (piRNAs) can be used only for diagnostic or prognostic purposes in this cancer and till now, no therapeutic effects are discovered for them but further investigations might improve our knowledge on whether they can be used for therapeutic purposes or not (Fig. 2). It worth to mention that a trial declared that combination of interleukin-6, piRNA, and a kind of micro RNA can lead to the transformation of cancer cells into CD4 + cells and therefore, it can be a useful procedure, in coming years, as combined therapy for colorectal cancer. Despite these advantageous, a problem stands in the way of detecting piRNA in patients’ serums: identifying the fragments of other ncRNAs instead of actual piRNAs. Recently, using miscellaneous-piRNAs (m-piRNAs) has been suggested as a solution to this problem but still, this method is not the definite answer [130].

Overall, the study of these RNAs in colorectal cancer is still in its infancy and there is a great number of obstacles in the way of establishing new piRNA-based prognostic or diagnostic procedures for being used in clinics.

## Data Availability

Not applicable.

## Abbreviations

NcRNA: Non-coding RNA
rRNA: Ribosomal RNA
tRNA: Transfer RNA
miRNA: Micro RNA
piRNA: PIWI- associated RNA
siRNA: Small interfering RNA
snoRNA: Small nuclear RNA
rasiRNA: Repeat-associated small interfering RNA
TE: Transposon element
CRC: Colorectal cancer
IBD: Inflammatory bowel disease
UTR: Untranslated region
RNAi: RNA interfering
RISC: RNA-induced silencing complex
LTR: Long terminal repeat
LINE: Long interspersed element
SINE: Small interspersed element
HP1a: Heterochromatin protein 1a
MM: Multiple myeloma
ccRCC: Clear cell renal cell carcinoma
HSP: Heat shock protein
TSS: Transcriptional start site
